# Separating Predicted and Perceived Sensory Consequences of Motor Learning

**DOI:** 10.1371/journal.pone.0163556

**Published:** 2016-09-22

**Authors:** Bernard Marius ‘t Hart, Denise Y. P. Henriques

**Affiliations:** Centre for Vision Research, York University, Toronto, Ontario, Canada; Ludwig-Maximilians-Universitat Munchen, GERMANY

## Abstract

During motor adaptation the discrepancy between predicted and actually perceived sensory feedback is thought to be minimized, but it can be difficult to measure predictions of the sensory consequences of actions. Studies attempting to do so have found that self-directed, unseen hand position is mislocalized in the direction of altered visual feedback. However, our lab has shown that motor adaptation also leads to changes in perceptual estimates of hand position, even when the target hand is passively displaced. We attribute these changes to a recalibration of hand proprioception, since in the absence of a volitional movement, efferent or predictive signals are likely not involved. The goal here is to quantify the extent to which changes in hand localization reflect a change in the predicted sensory (visual) consequences or a change in the perceived (proprioceptive) consequences. We did this by comparing changes in localization produced when the hand movement was self-generated (‘active localization’) versus robot-generated (‘passive localization’) to the same locations following visuomotor adaptation to a rotated cursor. In this passive version, there should be no predicted consequences of these robot-generated hand movements. We found that although changes in localization were somewhat larger in active localization, the passive localization task also elicited substantial changes. Our results suggest that the change in hand localization following visuomotor adaptation may not be based entirely on updating predicted sensory consequences, but may largely reflect changes in our proprioceptive state estimate.

## Introduction

When we initiate a movement, a copy of the motor command (efferent copy) is used to generate predictions about the motor outcome. Discrepancies between such predictions and the actual sensory feedback might be used to improve future performance [1,2]. This type of error-based motor learning is probably mediated by the cerebellum [3,4]. In order to fully understand this learning process, one would ideally have access to the efference copy involved in predicting sensory consequences.

Studies by Synofzik et al. [5] and Izawa et al. [6] have attempted to assess changes in predicted sensory consequences following visuomotor adaptation in both healthy individuals and those with cerebellar damage. They had participants make volitional movements with their unseen right hand to an arc spanning the first quadrant of the workspace. After returning the right hand to the home position, the participant indicated the location where they believed their hand crossed the arc either by controlling a cursor [5,7,8,9] or by moving the visible left hand to this location (“localization task”; [6,10]). They interpreted the observed shifts in localization following visuomotor adaptation as reflecting mainly updated predictions of visual sensory consequences.

Although visual feedback of the adapted hand was absent during localization in these studies, proprioceptive information was still available. Our lab has shown that visuomotor adaptation leads to both changes in reaching movements, and changes in proprioceptive estimates of current hand position. We call this change in felt hand position ‘proprioceptive recalibration’ [11,12,13,14,15,16,17], for a review, see: [18]. Changes in the proprioceptive state estimate appear to be independent of weights assigned to vision and proprioception [19]. Similar changes are observed following force-field adaptation [20] and gain modulation [21], or even when there is no motor component during training but only a discrepancy between visual and proprioceptive feedback of hand motion [22]. Finally, proprioceptive recalibration also lends itself to be incorporated in models of motor learning [23,24]. All these studies support the notion that proprioceptive recalibration is an integral part of visuomotor adaptation. However, most research on motor learning still places the greater emphasis on the *predictive* contribution and either gives *perceptual* changes minimal consideration or none at all. Hence our goal is to quantify the extent to which changes in hand localization reflect recalibrated proprioception, rather than updated predictions of sensory consequences.

To address this question, we replicated the paradigm used by Izawa et al. [6] where participants make a volitional hand movement with the adapted hand and then indicate the location where their unseen hand crossed an arc by tapping on a touchscreen (‘active’ localization). In order to disentangle the predicted from the actually perceived sensory consequences of a movement, we introduce a variation of the task where the robot manipulandum moves the participants’ hands instead, so that no efferent signals are available for generating a sensory prediction (‘passive’ localization), leaving only proprioception to generate a state estimate.

We also tested whether perceptual contribution to the changes in hand localization would be stronger if the trained hand remained at the end of the reach during localization (‘online’ localization). Izawa et al. [6] used ‘delayed’ localization (hand returned home) as a way to avoid this possibility. Thus, we included both an online and delayed version of the active and passive localization conditions to test this directly. However, our main goal was to determine the contributions of predicted versus perceived consequences in producing changes in hand localization following motor learning.

## Materials and Methods

### Participants

Twenty-one healthy, right-handed participants with normal or corrected-to-normal vision (mean age 24 years, range 18–38, 11 females) voluntarily took part in the experiment. All participants provided written, informed consent. Procedures were approved by the York Human Participants Review Sub-committee and were in accordance with the declaration of Helsinki.

### Setup

**Fig 1:**
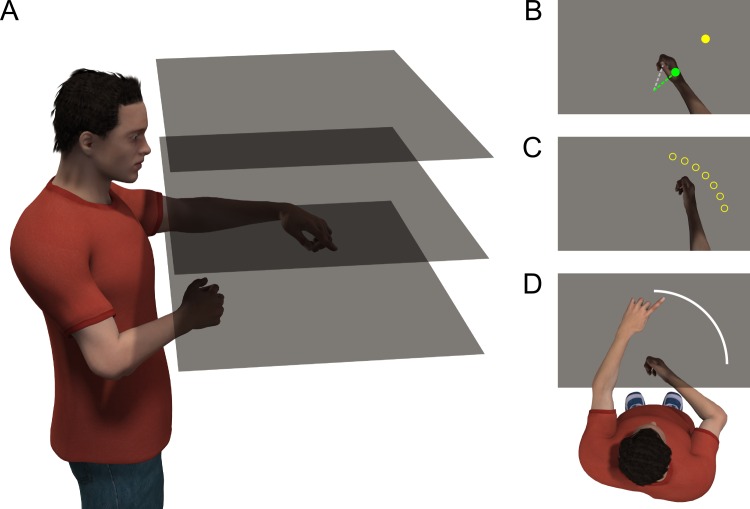
Setup and experimental design. **A:** Participants moved their unseen right hand with visual feedback on hand position provided through a mirror (*middle surface*) half-way between their hand and the monitor (*top surface*). A touchscreen located just above the hand was used to collect responses for the localization tasks and calibration (*bottom surface*). **B:** Training task. The target, shown as a yellow disc, is located 10 cm away from the home position at 45°. In the rotated training tasks, the cursor (shown here in as a green circle) represents the hand position rotated 30° relative to the home position. **C:** Display of the no-cursor reach task. Targets are located 10 cm away from the home position at 15°, 25°, 35°, 45°, 55°, 65°, and 75°, shown by the yellow circles here (only one was shown on each trial). While reaching to one of these targets, no visual feedback on hand position is provided. **D:** Localization task. The participants’ unseen, right hands moved out, and subsequently participants indicated the direction of the hand movement by pointing with their visible left hand at a location on a an arc on a touch screen.

With their right hand, participants held onto the vertical handle of a two-joint robot manipulandum (Interactive Motion Technologies Inc., Cambridge, MA, USA) such that their thumb rested on top of the handle. Visual stimuli were projected from a monitor (Samsung 510 N, 72 Hz) located 17 cm above the robotic arm. A reflective surface was mounted on a horizontal plane 8.5 cm above the two-joint robotic arm, midway between the manipulandum and the monitor, such that images displayed on the monitor appeared to lie in the same horizontal plane as that of the robotic arm (Fig 1A). A touch screen was mounted underneath the reflective surface, ~3.5 cm above the position of the thumb. Participants used their left hand–made visible with a small spot light–to indicate the location of their unseen right-hand, specifically the thumb, that rested on the handle of the manipulandum. For each task, the home position of the right hand was located about 20 cm in front of the participants, along the participants’ body midline.

### Procedure

All subjects completed a set of tasks in a specified order in two sessions performed on separate days, with the same order of tasks on each day (see Table 1). Each session started with a reach training task, followed by several localization tasks, as detailed below. In between the localization tasks there were blocks of additional training and no-cursor reaches. The experimentor first explained all tasks to the participants. Participants were prompted to perform training- and no-cursor reaches by a brief instruction on the screen. For training the instruction was “cursor”, for no-cursor it was “no cursor” and for all localization tasks the experimentor provided a verbal instruction.

**Table 1:** Block order.

	aligned	rotated
task	№ trials	
training	50	90
no-cursor reaches	-	21
training	-	60
active delayed	25	25
no-cursor reaches	21	21
training	10	60
21passive delayed	25	25
training21no-cursor reaches	10	60
active online	25	25
no-cursor reaches	21	21
training	10	60
passive online	25	25
no-cursor reaches	21	21

Blocks were performed from top to bottom, with two extra blocks in the rotated session. Trial numbers during training are larger in the rotated as compared to the aligned sessions. Passive localization tasks always follow the active localization tasks, since the robot moved the hand to the same arc-location in the passive condition as that produced by the participant in the active version. Before every localization task, training was reinforced, to minimize any decay in learning.

### Training

During training there was a single, visual target (a disc with a diameter of 1 cm), 10 cm away at 45° relative to the home position (Fig 1B). Participants were instructed to reach to the target as quickly and as accurately as possible. The hand was represented by a green, circular cursor, 1 cm in diameter. After placing the hand at the home position for 300 ms, the target appeared. Visual feedback of the hand position was continuously provided in the form of a cursor during the outward movement. A reach trial was complete when the center of the hand cursor overlapped with the target (i.e. the hand was within 0.5 cm of the target’s centre). Upon completion of the reach, both the cursor and target vanished and the participants moved their hand back toward the home position, along a constrained, straight path. That is, if participants tried to move outside of the path, a resistance force, a stiffness of 2 N/(mm/s) and a viscous damping of 5 N/(mm/s), was generated perpendicular to the path.

Aligned training consisted of 50 trials, with each top-up block containing 10 trials. During rotated training, visual feedback was gradually rotated around the home position, in clockwise steps of 0.75° per trial, until reaching 30°, where it remained for all subsequent trials and blocks. The rotated session began with a block of 90 training trials, and each of the top-up blocks contained 60 (see Table 1).

#### No-cursor reaches

Reach aftereffects were measured by having participants reach to targets in the absence of visual feedback. There were four blocks of no-cursor reaches in the aligned session and five in the rotated session, with the additional block being completed immediately after the initial reach training block. In each no-cursor block, participants reached to each of 7 targets: 15°, 25°, 35°, 45°, 55°, 65°, and 75° three times each, in random order, for a total of 21 reaches (Fig 1C). After the hand had moved out and been held in the same position for 500 ms, the target disappeared indicating that the trial was over. Participants then returned their hand to the home position along a constrained pathway, as in training.

#### Localization

In the localization tasks (Fig 1D), participants’ right hands moved out from the home position in a direction roughly between 0° and 90° up to an arc, 10 cm away from the home position. The right hand was then stopped by the robot, so that all reaches ended at 10 cm distance from the home position. Participants could see their left hand during the localization task, and used it to indicate, on the touch screen mounted above the manipulandum, the location where the movement of their unseen right hand had ended beneath the arc (cf. Izawa et al., 2012 [6]; participants were to point with the left hand to “where they believed their right hand crossed the circle”). In between localizations, the left hand was removed from the workspace and rested left of the touch screen. Crucially, there were four variations of this task. First, participants could move their own hand or the robot could move their hand to such a position. These are called the ‘active’ (spontaneous, self-initiated movement) and ‘passive’ (robot-generated movement) localization tasks. In the ‘passive’ localization tasks, the robot-generated movements were to the same endpoint angles that were recorded in the preceding ‘active’ task, but in a shuffled order. Second, either the participants could localize where the movement intersected with the arc around the home position while their hand was still at the endpoint of the movement (‘online’ localization) or after the hand had returned to the home position (‘delayed’ localization). Active and passive localization were each combined with online and delayed localization, yielding four different localization tasks. The active versions were performed first since the same movements were used to control the robot in the respective passive tasks. Each of these four localization tasks was done once after training with aligned visual feedback and once after training with rotated visual feedback.

### Analyses

Training reaches and no-cursor reaches were manually inspected for obvious movement errors, i.e. failure to perform the reach trial. Reach paths that could not be used for analyses were removed. Of the training trials, 4.7% were removed in aligned and 2.2% in rotated, for the no-cursor reaches, 7.7% of aligned and 0.5% of rotated reaches were removed. Of those retained, the endpoint angle was used for further analyses of the no-cursor-reaches, and the angle at the point of maximum velocity was inspected for the training reaches. For these points, the signed, angular difference between the actual hand position and the target was calculated. Aftereffects were calculated by subtracting the average angular differences between responses after aligned training from those after rotated training, within each combination of participant, task and target.

Before further processing, generic biases in localization were accounted for. For each participant, angular localization errors across the workspace and for all four aligned localization tasks (minus outliers defined as beyond ±3 standard deviations from the mean) were used to fit a second-degree polynomial. The remaining errors were fit with linear regression for online and delayed responses separately. The localization errors predicted by this simple model given a localization response, were subtracted from touch screen response angles in both the aligned and rotated localization tasks. The data in each localization task was then binned according to the angle of the endpoint of the unseen hand movement relative to the home position. Bins were 10° wide and centred on the 7 targets used in the no-cursor reach task (15°, 25°, 35°, 45°, 55°, 65°, and 75°). Within each bin the average deviation of the localization of the right hand with the touch screen from the actual endpoint of the hand was calculated in degrees. This was done separately within each participant, for each of the eight localization tasks; aligned vs. rotated training, active vs. passive and delayed vs. online. Some participants did not make any reaches in some bins, though this is mostly restricted to the 15° and 25° bin. Similarly to the no-cursor reaches, we subtracted the localization angles after aligned training from those after rotated training, to obtain an estimate of the effect of training on localization of the hand.

#### Data analysis

For all ANOVAs we used a linear mixed effect model, (instead of a general linear model) as it is robust against empty cells, which occur in the localization data. Most of these models included *participant* (1–21) and either *target* (for reach aftereffects) or *bin* (for localization; 15°, 25°, 35°, 45°, 55°, 65°, and 75°) as random effects. There were two exceptions: the ANOVAs testing aftereffects as well as the localization changes across the workspace included *target* and *bin*, respectively, as a fixed effect. For both the no-cursor reach endpoint angles as well as the localization angles, we first tested if there was an effect of training on these angles to begin with, by comparing the responses after aligned training with those after rotated training. For further analyses we subtract the responses after aligned training from those after rotated training as a measure of training-induced change and compare the different conditions on this measure.

To test whether training with a rotated cursor led to changes in no-cursor reaches (i.e. if there were reach aftereffects), we ran a two-way ANOVA using a linear mixed effects model on the angular deviation of the reach endpoint from target across all iterations of the no-cursor reach tasks with *training* (aligned or rotated) and *target* as fixed effect. To see if reach aftereffects measured after localization changed over time in the rotated session, we took the angular deviations in each iteration of the no-cursor block, and subtracted from this the average angular deviation across all blocks in the rotated session, separately for each participant and target. This way, we obtained an estimate of the training-induced change on no-cursor reach endpoint angle for the five iterations of no-cursor reach blocks in the rotated session. We then did an ANOVA on a linear mixed effect model of the shift in the angular bias, using *iteration* (1–5) and *target* as fixed effects. Furthermore, we inspected decay of reach aftereffects by first calculating the average response for each trial number within a block on the central three targets (35°, 45° and 55°). We then bootstrapped the 95% confidence interval across participants on the average effect on the central three targets with 100,000 iterations in the 4 blocks of no-cursor reaches following a localization task in the rotated session. We then compared this with the decay of the effect in the no-cursor reach block immediately following training.

To see if rotated-cursor training had any effect in any of the four localization tasks, we ran a one-way ANOVA on the average difference between the hand angle and the localized angle in every bin, for each of the tasks separately, with the fixed effect *training* (aligned or rotated). To test if delayed or online localization either combined with active or passive movements responded differently to training, we ran a three-way ANOVA on a model with *training*, *moment of localization* and *movement type* as fixed effects. Finally, to see if the effects of *movement type* and *moment of localization* on localization were different across the workspace, we ran a three-way ANOVA on the difference between localization responses after rotated and after aligned training (i.e., the training-induced shift in localization), with *bin*, *movement type* and *moment of localization* as fixed effects.

So far, we assumed that the effects of training on proprioception and predicted sensory consequences were added in the active conditions. However, if proprioception and predicted sensory consequences were integrated in a Bayesian manner, then the contribution of proprioception to the overall state estimate depended on it’s accuracy relative to the accuracy of predicted sensory consequences. This accuracy can be expressed as the inverse of the variance [25], and such an approach has been used to model the integration of proprioception with actual visual consequences [26, 27, 28]. If efference-based predictions of hand location were much more accurate than proprioception-based localization and the two signals were integrated in a Bayesian manner, then the variance in the active localization conditions should be lower than the variance in the passive localization conditions for all participants. We tested this possibility using a binomial exact test on all data, as well as separately for all four combinations of online and delayed localization on the one hand and aligned and rotated training on the other hand. We also tested if the same participants show this hallmark of Bayesian integration in the aligned and rotated subsets separately for the delayed as well as the online localization using two Fisher exact tests.

Data preprocessing was implemented in Python 2.7 (using numpy, scipy, pandas and various other modules) and statistical analysis in R 3.0.2 [29] (using nlme and other packages).

## Results

We set out to quantify to what extent people rely on predictions of sensory consequences or on proprioception when they locate where their unseen hand moved. Our participants did several localization tasks as well as no-cursor reaches after training with both aligned and rotated visual feedback.

### Reach Aftereffects

**Fig 2:**
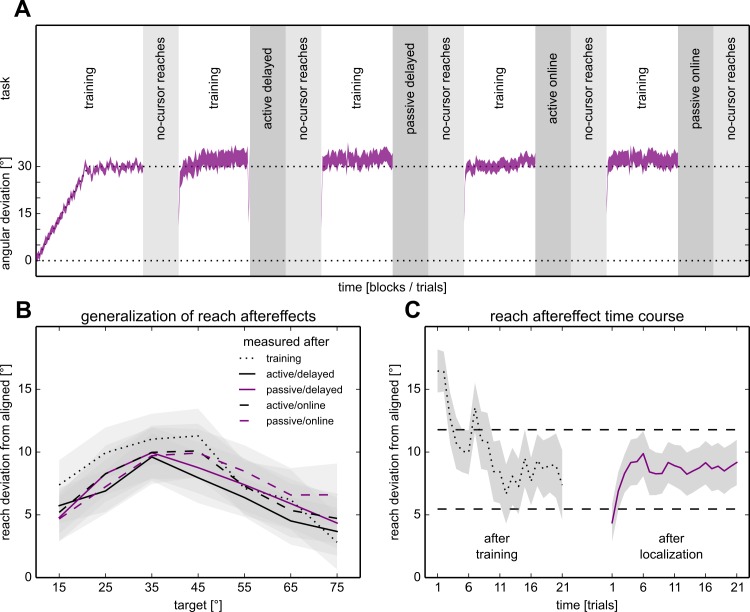
Time course, and reaching results, of the experiment. **A, top:** task/block order. Each session started with a longer training block. The four localization tasks were performed after a block of training and before a no-cursor reach block. The order was similar for the aligned and rotated sessions, but the rotated sessions had an extra no-cursor reach block and training block after the initial training block. **A, bottom:** Hand movement direction while reaching during the rotated session. The average angle at the point of peak velocity over trials (purple area in the training blocks denotes the average across participants ± SEM) shows that participants adapted their reaching movements to rotated visual feedback in step with the gradually introduced rotation of visual feedback. Reach aftereffects are shown in the no-cursor reach blocks. The curves go from the target at 15° (at the left side of the curve) to the target at 75° in order, and the circles denote the aftereffects at the 45° target, the only target used during training. **B:** Generalization of reach aftereffects. The difference between the average reach endpoint angles in each of the no-cursor blocks done after rotated training, corrected for the average reach endpoint angles across all the no-cursor blocks done after aligned training. Gray areas represent the standard error of the mean. Note that the effect is strongest at the trained target, and decreased for targets further away. Also, the reach aftereffects measured immediately after training are slightly larger than those in the other blocks. **C:** Time course of reach aftereffects. *Left*: On the very first trial following training, reach aftereffects are about twice as large as at the end of that block. *Right*: Except for some initial errors, the reach aftereffects measured on the central three targets seems stable in the no-cursor reach blocks following localization. Gray areas indicate standard error of the mean, dashed lines indicate the 95% confidence interval for responses in the blocks after localization.

As can be seen in Fig 2A, participants adjust their reach directions (shown in purple) when faced with a gradually introduced visuomotor rotation, suggesting that they adapt to the visuomotor rotation. This also leads to significant changes in no-cursor reaches, as illustrated by the “generalization” curves in Fig 2B (main effect of *training* (F(1,260) = 24.17, p < .001). Although the size of the aftereffects appears to decrease for novel directions further from the trained direction, the effect of *training* does not significantly interact with *target* direction (F(6,260) = 2.020, p = .063). So although aftereffects appear strongest around the trained direction (45°), generalization is broad enough to elicit aftereffects across most of the 0°–90° workspace.

Additionally, we wanted to test if reach aftereffects changed over the course of the rotated session, as this may impact the results in the localization tasks. This was not the case: there is no effect of *iteration* (F(4,680) = 1.68, p = .153), nor any interaction of *target* and *iteration* (F(24,680) = 0.609, p = .930). Therefore, the pattern and magnitude of visuomotor adaptation is comparable for all localization tasks. To see if there was any decay of effects within the no-cursor reach blocks, we averaged the effects for the central three targets, for each trial and participant, across the four no-cursor reach blocks that followed a localization task (Fig 2C, right) as well as the one that immediately followed training (Fig 2C, left). When the no-cursor reaches succeeded a localization task, participants apparently needed to re-acquaint themselves with the task in the first few trials and then proceeded to produce constant responses. In the no-cursor reach block that immediately followed training, the effect is larger at first but stayed within the 95% confidence interval after 8 trials (about one third of the block). Hence, decay seems to play only a minor role in our data.

### Localization

**Fig 3:**
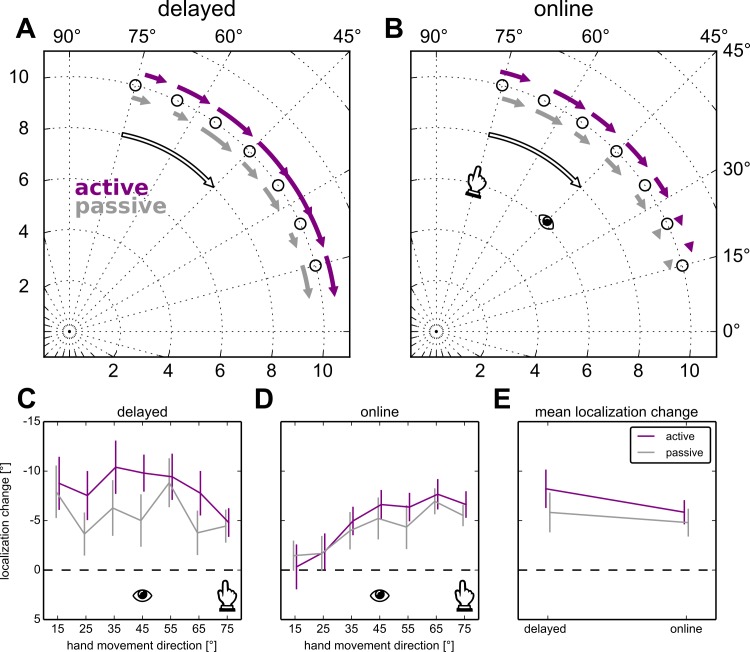
Localization results. The change in the touchscreen responses in the four variations of the localization task, using 10° bins centered on the reach targets (circles) in the no-cursor reach block. Active localization is shown in purple, passive localization in gray. The eye-icons illustrate the direction of the target during training, and the hand icons illustrate the direction of movements required to hit the target with 30° rotated visual feedback. **A,C:** Delayed localization, **B,D:** Online localization. **A,B:** The beginning and end of the arrows show the average deviation from the true reach angle in that bin before and after visuomotor adaptation, respectively. The open arrow illustrates the visuomotor rotation. **E:** The average change in the direction of hand localization across bins and participants.

As can be seen in Fig 3A–3E, adapting to a visuomotor rotation leads to a change in hand localization for each of the four localization tasks, as verified by four one-way ANOVAs that show an effect of rotated versus aligned *training* (all p < .0001). This main effect of *training* persists when we run a three-way ANOVA also including *movement type* and *moment of localization* as fixed effects (F(1,943) = 272.25, p < .0001). The effect of *training* varies with *movement type* (F(1,943) = 5.87, p = .016) and with *moment of localization* (F(1,943) = 7.08, p = .008), but there is no interaction between *movement type* and *moment of localization* (F(1,943) = 1.14, p = .285) and no three-way interaction (F(1,943) = 1.38, p = .240). Specifically, changes in active localization (online: 5.8°; delayed: 8.2°) are close to 30% larger than those in passive localization (online: 4.8°; delayed: 5.8°), as illustrated in Fig 3E. This pattern of results suggests that changes in indicating unseen hand location following visuomotor adaptation likely reflects mostly plasticity in the proprioceptive estimates (over 75%) rather than updated predictions of hand position. While changes in perceived hand position are likely responsible for most of the change in indicating hand direction, we find no evidence for a larger contribution of proprioceptive recalibration to localization when the hand remains at the reach endpoint as compared to when it moved back to the home position first (see also Fig 3E).

The differently sized effects across the workspace as seen in Fig 3C and Fig 3D suggest different generalization patterns for online and delayed localization. When we test the difference between localization following rotated and aligned training, and include *bin* as a fixed effect along with *movement type* and *moment of localization*, we find that *bin* interacts with *moment of localization* (F(6,473) = 2.91, p = .009). As might be expected, there is a main effect of *movement type* (F(1,473) = 11.36, p = .0008) and of *moment of localization* (F(1,473) = 9.50, p = .002), but there’s no main effect of *bin* (F(6,473) = 1.59, p = .149) and no other effects (all p>.335). Hence, the shape of the pattern of change across the workspace is different between online and delayed localization, but this pattern is not different for active and passive movements.

To see if Bayesian integration of perceived and predicted hand position makes more sense than a simple addition of the shifts, we compare the variance of the errors in active and passive localization in all four conditions, using all reaches where the actual hand was between 10° and 80°. If efference-based predictions are integrated with proprioceptive feedback, there should be a lower variance in the active conditions as compared to the passive conditions. Across every participant, for aligned/online, rotated/online, aligned/delayed and rotated/delayed this is the case for 47 out of 84 datasets, which is not different from chance according to a binomial exact test (p = .326). If we test whether the number of participants with lower error variance in active, as compared to passive movements deviates from chance (50%) in online/aligned (12/21, p = .6636), online/rotated (12/21, p = .6636), delayed/aligned (10/21, p = 1.) and delayed/rotated (13/21, p = .3833; no correction for multiple comparisons) this doesn’t change. It may still be the case that a subset of participants is able to use Bayesian integration, whereas others are not. We test this with two Fisher exact tests. These are performed on a contingency table that counts how many participants have a lower variance in the active localization as compared to passive localization in rotated localization only, delayed localization only, both or neither. For both the online datasets (odds ratio: 0.517, p = .661) as well as the delayed datasets (odds ratio: 0.863, p = 1.) this is not the case. These results imply that the variance in active and passive conditions are about equal and that their relative amplitude doesn’t vary systematically with participant or with condition. While these results seem to contradict a Bayesian integration of proprioception with efference-based predictions, we can’t exclude the possibility. However, if the two cues are integrated in a Bayesian fashion, the absence of a detectable decrease in variance in our dataset suggests that the variance of efference-based predictions of sensory consequences is at least as large as the variance of proprioception. Thus, the weight of proprioception in the integrated signals should be as large as or larger than the weight of efference-based predictions in the integrated state estimate. Regardless of whether proprioceptive signals and predictions are integrated in a Bayesian manner to arrive at an estimate of hand position or whether they are simply added, the data show that the role of proprioception in visuomotor rotation adaptation is clearly non-neglible.

## Discussion

The aim of this study is to quantify to what extent the changes in hand localization following visuomotor adaptation may actually depend on (recalibrated) proprioception, rather than predicted sensory consequences. To this end, we replicate a task intended to measure changes in predicted sensory consequences [6,10], that is similar to a task used in other studies [5,7,8,9]. In brief, before and after adapting reaches to a visuomotor rotation, participants indicate the location of their unseen right hand–with their visible, non-adapted, left hand–when a right-hand movement is planned and executed by the participant themselves (i.e. with predicted sensory consequences) or when the right hand is moved by the robot toward the same location (in the absence of predicted sensory consequences). We find that changes in localization following passive movements are significant, and amount to over two-thirds of those in active movements. Given that the variance in localization response errors doesn’t decrease with the availability of an additional source of information in the active localization tasks, alternative explanations relying on a Bayesian integration account of the data seem unlikely. In other words, the changes in localization following motor learning are substantially influenced by a change in perceived hand position. Thus, while motor learning does likely lead to updating of forward models, our results illustrate the inherent difficulty in disentangling changes in predicted sensory consequences from changes in proprioceptive state estimates.

We also test the assumption that online localization would be more strongly influenced by proprioception as the hand is still at the target site (online), which is not the case when it is removed from the reach endpoint prior to localization (delayed) as was done by Izawa et al. [6] and Synofzik et al. [5]. However, while the pattern of generalization was different for online and delayed localization, we found that the moment of localization is not as relevant as previously thought for the overall amplitude of the effect. This is consistent with our previous findings that a short delay before localizing the position of a robot guided hand only leads to a decrease in precision, but not in accuracy [30]. Furthermore, the magnitude of proprioceptive recalibration is not modulated by the precision of proprioception, such as in older compared to younger adults [12]. Similarly, Block and Bastian [19] found no relationship between weights assigned to vision and proprioception and the resulting changes in hand proprioception following visuomotor adaptation. Given that the current results suggest that visuomotor adaptation affects both the perceived as well as the predicted estimates of hand motion, it may be that removing the target-hand from the final position (where current proprioceptive information would be available) may simply not provide any immunity to the effect that visuomotor adaptation seems to have on perceived estimates of hand position.

### Predicted and perceived hand location

Our findings suggest that people–perhaps unintentionally–use the perception of their completed movement when reporting the planned movement, as has been suggested earlier [5,6], although without accounting for it. Proprioception is still available in the localization task, and we have shown that proprioceptive estimates of hand position are recalibrated during visuomotor adaptation under various conditions [11,13,14,15,16,17,22,31,32,33]. Proprioceptive recalibration has also been demonstrated in force-field adaptation [20] and in gain modulation [21]. The use of robot-guided movements in our experiments is meant to avoid the efferent signals that could lead to the predictive component that Izawa et al. [6] and Synofzik et al. [5] were trying to capture in their related studies. Cameron et al., [20] have used EMG recordings to show this to be an effective method to prevent participants from generating goal-directed movements, and hence efferent signals necessary for prediction. Here we use both active movements to assess changes in predicted sensory consequences, as well as passive movements to isolate the purely perceptual component of “predictive” responses. As far as we know, one previous study has found comparable results, albeit using a one-dimensional movement and gain modulation of visual feedback [21]. The contribution of proprioceptive recalibration they find (between ¼ and ⅓) is smaller than what we find here, but nevertheless far from negligible. Interestingly, this study also investigates passive “exposure” training [22,33,34]. Hand movements in exposure training generate the same visual feedback relative to the actual hand position as in regular training, so that discrepancies between vision and proprioception are preserved. However, since the robot moves the hand such that the cursor reaches the target without error, expected sensory consequences can’t be updated in exposure training. The results with exposure training confirm their findings with ‘regular’ training, as well as earlier findings from our lab. That is, in both the active and passive exposure session, they find training induced changes on all dependent variables. There are no appreciable differences between the changes in variables assessing proprioception alone, depending on the type of training. On the other hand, 24% of the adaptation score for active perception trials and 35% of the adaptation score for active target trials can be explained by proprioceptive recalibration. However, despite the thorough work by Cameron et al. [21], vision-only models and explanations of motor learning remain dominant.

Both papers we based our experiment on [5,6] explicitly state their conclusions in terms of predicted visual consequences only. There are some differences between those paradigms and ours, such as the type of reaching movement, of which it is known they make no difference [3]. The absence of training targets in one paradigm [5] could differentiate it from motor learning tasks, but the same target-less task is still explicitly used as a motor learning paradigm elsewhere [9]. Despite the differences, we find very similar patterns of results in our active condition, marking all three studies as highly comparable, visuomotor rotation paradigms. Hence, the results in our passive condition should highlight the relevance of proprioceptive recalibration to studies on motor learning. One might even wonder if there is a proprioceptive component in the changes in predicted sensory consequences due to motor learning. To what extent changes in predicted proprioceptive consequences play a role in the forward model is largely unknown. Yet, most recent studies on motor learning seem to implicitly assume that vision is the only relevant modality. In contrast, our results here clearly indicate that the contribution of proprioceptive recalibration to changed state estimates after visuomotor rotation adaptation are non-negligible and distinct from both the contributions of vision or efference-based predictions of sensory consequences to motor learning.

### The role of the cerebellum

A combination of changes in perceptual and prediction-based contributions to estimates of hand position can explain the reduced, yet still significant mislocalization of hand position following visuomotor adaptation in cerebellar patients in the studies by Synofzik et al. [5] and Izawa et al. [6]. The localization shifts for patients in those studies were about half of those for healthy controls, but were still significant. When we substitute the usual visuomotor rotation training with mere exposure to a discrepancy between visual and proprioceptive feedback on hand position, the resulting proprioceptive recalibration is comparable [21,22,33,34]. This process seems to be intact in cerebellar patients [34,35]. Hence the remaining changes in localization found by Synofzik et al. [5] and Izawa et al. [5] may reflect normal proprioceptive recalibration (occurring outside the cerebellum, perhaps in the posterior parietal cortex, e.g., Shadmehr et al. [4]). The reduction in the shift, however, may reflect the loss of the predictive contribution to estimating hand position, or perhaps even deficits in combining predictive and perceived contributions. This difficulty in distinguishing between these two possibilities also arises in studies investigating the role of cerebellum more specifically on state estimation [36,37]. Interestingly, Synofzik et al. [5] found no visuomotor learning (i.e., reach aftereffects) in their patient group, whereas Izawa et al. [6] did, but both studies still found comparable effects in their respective versions of the localization task. This can be explained if the mechanism for proprioceptive recalibration is distinct from the mechanism for visuomotor learning [34,35] and both contribute separately to the measurements in localization.

### Conclusion

We found that changes in limb localization following visuomotor adaptation are not mostly due to updated predictions of sensory consequences, but also substantially reflect changes in sensory-based state estimates. These results are consistent with our theory that recognizes that sensory plasticity likely plays a much larger role in motor learning than usually assumed.
