# Correction: Separating Predicted and Perceived Sensory Consequences of Motor Learning

**DOI:** 10.1371/journal.pone.0168825

**Published:** 2016-12-19

**Authors:** Bernard Marius ‘t Hart, Denise Y. P. Henriques

There are errors in Table 1. The shading is misaligned and certain numbers are out of order. Please see the correct version of Table 1 here:

**Table 1:** Block Order. Blocks were performed from top to bottom, with two extra blocks in the rotated session. Trial numbers during training are larger in the rotated as compared to the aligned sessions. Passive localization tasks always follow the active localization tasks, since the robot moved the hand to the same arc-location in the passive condition as that produced by the participant in the active version. Before every localization task, training was reinforced, to minimize any decay in learning.

task	aligned	rotated
	№ trials	
training	50	90
no-cursor reaches	-	21
training	-	60
active delayed	25	25
no-cursor reaches	21	21
training	10	60
passive delayed	25	25
no-cursor reaches	21	21
training	10	60
active online	25	25
no-cursor reaches	21	21
training	10	60
passive online	25	25
no-cursor reaches	21	21

## References

[1] ‘t Hart BM, Henriques DYP (2016) Separating Predicted and Perceived Sensory Consequences of Motor Learning. PLoS ONE 11(9): e0163556 doi: 10.1371/journal.pone.0163556 27658214 10.1371/journal.pone.0163556 PMC5033392

